# Phase I dose-escalation oncology trials with sequential multiple schedules

**DOI:** 10.1186/s12874-021-01218-9

**Published:** 2021-04-14

**Authors:** Burak Kürsad Günhan, Sebastian Weber, Abdelkader Seroutou, Tim Friede

**Affiliations:** 1 Department of Medical Statistics, University Medical Center Göttingen, Göttingen, Germany; 2 Novartis Pharma AG, Basel, Switzerland

**Keywords:** Phase I dose-escalation trials, Multiple treatment schedules, PK models, Bayesian statistics

## Abstract

**Background:**

Conventional methods for phase I dose-escalation trials in oncology are based on a single treatment schedule only. More recently, however, multiple schedules are more frequently investigated in the same trial.

**Methods:**

Here, we consider sequential phase I trials, where the trial proceeds with a new schedule (e.g. daily or weekly dosing) once the dose escalation with another schedule has been completed. The aim is to utilize the information from both the completed and the ongoing schedules to inform decisions on the dose level for the next dose cohort. For this purpose, we adapted the time-to-event pharmacokinetics (TITE-PK) model, which were originally developed for simultaneous investigation of multiple schedules. TITE-PK integrates information from multiple schedules using a pharmacokinetics (PK) model.

**Results:**

In a simulation study, the developed approach is compared to the bridging continual reassessment method and the Bayesian logistic regression model using a meta-analytic-predictive prior. TITE-PK results in better performance than comparators in terms of recommending acceptable dose and avoiding overly toxic doses for sequential phase I trials in most of the scenarios considered. Furthermore, better performance of TITE-PK is achieved while requiring similar number of patients in the simulated trials. For the scenarios involving one schedule, TITE-PK displays similar performance with alternatives in terms of acceptable dose recommendations. The R and Stan code for the implementation of an illustrative sequential phase I trial example in oncology is publicly available (https://github.com/gunhanb/TITEPK_sequential).

**Conclusion:**

In phase I oncology trials with sequential multiple schedules, the use of all relevant information is of great importance. For these trials, the adapted TITE-PK which combines information using PK principles is recommended.

## Background

Phase I trials constitute the first step in investigating the safety of potentially promising therapies in humans [1]. In many disease areas, phase I trials are conducted in healthy subjects which are not expected to benefit from the therapy. Phase I trials in healthy subjects include single and multiple ascending trials [2]. In the single ascending dose trials, the effects of a single dose on subjects are investigated, whereas multiple ascending dose trials investigate the effects of multiple doses. In life-threatening diseases such as in oncology, however, patients with few therapeutic options are recruited for a phase I trial, since therapies are usually highly toxic [3]. The main assumption is that both the probability of toxicity and the probability of efficacy are increasing with dose. Thus, the drug is expected to have very little efficacy at low doses [3]. In this paper, we focus on phase I trials in oncology.

Phase I trials in oncology traditionally enroll small cohorts of patients who are treated in treatment cycles. The observed toxicities are classified into dose-limiting toxicities (DLT) and non-DLT. Each time a cohort completes the first cycle at a given dose level, the available data are assessed to decide how the trial proceeds. The main aim is to identify the maximum tolerated dose (MTD). The MTD can be defined as the dose level at which the probability of DLT is closest to a target probability, usually 25% or 30% [4]. Typically, the estimation of the MTD is based on the toxicity data of the first cycle only. The setup of a phase I trial includes pre-specified doses to be evaluated, a starting dose which is considered safe, cohort size, maximum sample size and other stopping rules. If all doses have very low DLT probabilities, the dose which has DLT probability closest to the target probability can be declared as the MTD, after the maximum sample size is reached. Furthermore, the starting dose of the trial is traditionally determined one tenth of the lethal dose for mice or one sixth the highest non-severely toxic dose in a more sensitive species such as monkeys [5].

Standard methods for phase I dose-escalation trials in oncology include algorithm-based methods such as 3+3 designs [6] and model-based methods such as the continual reassessment method (CRM) [7]. The CRM uses a statistical model to estimate the relationship between the dose and the probability of DLT, which informs dose-escalation decisions. The Bayesian logistic regression model (BLRM) [4, 8] is a two-parameter version of the CRM which utilizes the escalation with the overdose control (EWOC) [9] criterion. The EWOC criterion aims to reduce the risk of overdosing patients by choosing doses with a posterior probability of being above the true MTD lower than a feasibility bound.

In addition to the dose administered, the frequency of administration, known as the schedule, is a crucial part of a treatment plan of any phase I trial. In practice, sometimes it is required to investigate multiple schedules, e.g. a dose given once a day or an adequately larger dose given once a week. Hence, the probability of DLT for each patient is a function of both the dose and the schedule. Simultaneous investigation of dose and schedule within a phase I trial has gained some attention in the literature. In such trials, the doses and the schedules are altered for different cohorts of patients within the same trial. Methods for simultaneous investigation of dose and schedule combination include a Bayesian time-to-event model by Braun et al. [10] and the partial order continual reassessment method by Wages et al. [11]. Recently, Günhan et al. [12] proposed an alternative dose-schedule finding method, a Bayesian time-to-event pharmacokinetics model (TITE-PK), which uses pharmacokinetics (PK) principles. Unlike other phase I methods, TITE-PK makes use of an exposure-response model that is often more informative than a standard dose-response model. TITE-PK models the relationship between time-to-first DLT and an exposure measure of the drug obtained by a *pseudo*-PK model in a Bayesian model-based approach. TITE-PK has been shown to have desirable operating characteristics in terms of finding an acceptable dose and schedule simultaneously in simulation studies [12].

In this paper, we consider an alternative phase I design in which multiple treatment schedules are investigated sequentially, rather than simultaneously. The schedules are denoted by *S*_*i*_ where *i*=1,2,…,*k*. The sequential multiple schedule design proceeds as follows. In the first step, cohorts of patients are enrolled with *S*_1_ and the trial is continued until the MTD is declared for *S*_1_. In the second step, the trial continues with schedule *S*_2_ and the starting dose can be informed from the *S*_1_. Dose-escalation decisions are informed by utilizing information from both schedules *S*_1_ and *S*_2_. That is, data from both the completed schedule *S*_1_ and the ongoing schedule *S*_2_ are integrated. Once the MTD for the Schedule *S*_2_ is determined, the trial can continue with schedule *S*_3_ and so on. In other words, the MTD for schedule *S*_*i*_ declared in the *i*th schedule of the phase I trial.

A sequential phase I trial with different strata, where strata may correspond to different patient populations, formulations, or treatment schedules etc., also called a bridging trial, was considered by Liu et al. [13] among others [14–16]. Liu et al. [13] introduced the bridging CRM (B-CRM) to borrow information from different strata. B-CRM takes into account potential heterogeneity between different strata using a Bayesian model averaging approach. Neuenschwander et al. [14] suggest the use of BLRM with a meta-analytic-predictive (MAP) prior [17] approach (BLRM-MAP) to take advantage of the completed step of the trial with different strata.

Borrowing approaches are based on discounting the existing information at the cost of increasing the needed sample size to achieve an acceptable performance in a new trial. Here we suggest the use of a modelling approach based on PK principles in order to increase the statistical efficiency. Therefore, we adapted the TITE-PK to design and analyze sequential phase I trials with multiple schedules. In the first step, TITE-PK is used to inform dose-escalation decisions for schedule *S*_1_ until the MTD is declared or the trial is stopped. In the next step, TITE-PK models the data from both the completed (*S*_1_) and the ongoing (*S*_2_) steps of the trial directly, but only recommending doses for Schedule *S*_2_. TITE-PK can be used for any number of schedules. We investigate the operating characteristics of TITE-PK for phase I trials with one schedule and sequential phase I trials with multiple schedules through simulations. We provide simulation results comparing the performance of TITE-PK to CRM and BLRM for phase I trials involving one schedule and to B-CRM and BLRM-MAP for sequential phase I trials involving multiple schedules. This paper builds on the previous work by Günhan et al. [12], and offers two main contributions. Firstly, we adapt TITE-PK to phase I dose-escalation trials with sequential (rather than simultaneous) multiple schedules. Secondly, we apply TITE-PK for a standard phase I dose-escalation trial, that is phase I trial involving a single schedule only.

### Illustrative example: everolimus trial

Everolimus (RAD001) is an oral inhibitor of mammalian target of rapamycin, that has been developed as an antitumor agent [18]. Everolimus is approved by the US FDA to treat various conditions including certain types of pancreatic cancer and gastrointestinal cancer [18] and certain type of tuberous sclerosis [19]. The elimination half-life and the absorption rate of everolimus for cancer patients were reported as 30 (hours) and 2.5 (1/hours), respectively [20]. Everolimus was included in a phase Ib trial in combination with standard of care (etoposide and cisplatin chemotherapy) to identify a feasible dose and schedule in the treatment of small cell lung cancer (ClinicalTrials.gov identifier: NCT00466466) [21]. Note that this trial did not include the initial human exposure to everolimus, since everolimus was investigated for different types of diseases, previously. Hence, this trial was an example of an effort for drug re-purposing.

The everolimus trial was open-label and multi-centered. Patients were assigned alternately to either weekly or daily schedules of everolimus in treatment cycles of 21 days. In the everolimus trial, doses in both schedules were escalated simultaneously and analysed separately from one another. A Bayesian time-to-event model [22] was used to inform the dose-escalation decisions. The final data can be obtained from the supplementary material of Besse et al. [21]. The dataset is displayed in Table 1. All DLT were reported at day 15. Based on investigator and medical monitor opinion, 2.5 mg with daily schedule was identified as the MTD [21].

**Table 1:** Data of the everolimus trial. The treatment schedules which are used, the doses which are administered in mg, number of patients, and number of DLT are given

Schedule	Dose	Number	Number
	(mg)	of patients	of DLT
Weekly	20.0	5	0
Weekly	30.0	13	4
Daily	2.5	4	2
Daily	5.0	6	3

We used this trial to illustrate the TITE-PK approach for sequential designs, because (1) the trial is a phase I dose-escalation trial in oncology, (2) the trial evaluated two different schedules (weekly and daily dosing), and (3) the large number of DLT allows a good assessment on the performance of the TITE-PK. We will analyse the final dataset as if the trial had been conducted sequentially, specifically assuming *S*_1_ is weekly schedule and *S*_2_ is daily schedule.

This paper is organized as follows. In the following section, we describe statistical methods for phase I trials with sequential multiple schedules. We review the BLRM and CRM. Then, we develop TITE-PK for sequential investigation of multiple schedules. The performance of TITE-PK and comparators are studied in simulations, and in the everolimus example. We close with a brief discussion and some conclusions.

## Methods

### The Bayesian logistic regression model (BLRM)

The models described in this section follow the BLRM and BLRM MAP described by Neuenschwander et al. [8]. The BLRM is a logistic regression model in the logarithm of a standardized dose. For dose *d*, the number of patients with a DLT (*r*_*d*_) in a cohort of size *n*_*d*_ are assumed to be binomially distributed

$$\begin{array}{*{20}l} r_{d} \sim \text{Bin}(\pi_{d}, n_{d})  \end{array} $$

with DLT probabilities (*π*_*d*_) and two parameters (*α*_1_ and *α*_2_)

$$\begin{array}{*{20}l} \text{logit}(\pi_{d}) = \, \log(\alpha_{1}) + \alpha_{2} \, \log(d/d^{*}),  \end{array} $$

where *d*^∗^ is the reference dose used for standardization of the dose. At the reference dose, the odds of the DLT are *α*_1_. Thus, the reference dose is critical in choosing a prior for *α*_1_. The reference dose is defined such that the reference dose *d*^∗^ is set to the anticipated MTD at which an odds of 1/2 is used as mean for the *α*_1_ prior.

To inform the dose-escalation decisions, the posterior distribution of the DLT probability *π*_*d*_ is used. The DLT probabilities are classified into three categories as follows

(i) *π*_*d*_<*x* Underdosing (UD)
(ii) *x*≤*π*_*d*_≤*y* Targeted toxicity (TT)
(iii) *π*_*d*_>*y* Overdosing (OD)

Escalation or de-escalation decisions are informed using the overdosing probability of dose *d*, *P*(*π*_*d*_>*y*). The EWOC criteria is fulfilled, if *P*(*π*_*d*_>*y*) is smaller than the pre-specified feasibility bound, *a*, which was recommended as 0.25 by Babb et al. [9]. Following this advice, we use *a*=0.25 throughout the manuscript. Among the doses which fulfill the EWOC criterion, the highest dose is recommended for the next cohort. Once the maximum sample size is reached, the highest dose among the doses satisfy EWOC criterion is declared as the MTD.

In the absence of relevant historical data, Neuenschwander et al. [8] suggest the use of weakly informative priors (WIPs) for *α*_1_ and *α*_2_ instead of flat priors. There are two problems regarding flat priors. Firstly, no formal analysis is possible until one DLT is observed in the trial, since the posterior is proportional to the likelihood. Secondly, flat priors on the *α*_1_ and *α*_2_ result in U-shaped priors for the DLT probabilities [4]. Their suggested WIP is a bivariate normal distribution $(\log (\alpha _{1}), \log (\alpha _{2})) \sim \mathcal {N}(\mathbf {m}, \mathbf {S})$ with means $\phantom {\dot {i}\!}(m_{1} = \text {logit}(\pi _{d^{\ast }}), m_{2} = 0)$, standard deviations (*σ*_1_=2,*σ*_2_=1), and the correlation *ρ*=0 [8]. Here, $\phantom {\dot {i}\!}\pi _{d^{\ast }}$ is the anticipated DLT probability at the reference dose. A derivation of the suggested WIP for the BLRM can be obtained using quantiles from minimally informative uni-modal Beta distributions [4]. An extension of the BLRM is used to incorporate different schedules in a phase I trial, which we describe in the following.

#### The BLRM MAP

The BLRM with a meta-analytic predictive (MAP) approach [23, 24] can be used for informing dose-escalation decisions in a phase I study with multiple schedules. Hereafter, we refer this method as the BLRM MAP. In the BLRM MAP approach, the doses from the first schedule (*S*_1_) are re-scaled so that two sets of doses from different schedules are comparable. For example, if *S*_1_ is weekly dosing and *S*_2_ is daily dosing, the doses from *S*_1_ are divided by 7. This ensures that the respective nominal dose in each schedule results in the same cumulative dose. Then, a meta-analytic-predictive (MAP) prior is derived using the data of the *S*_1_ assuming some between-schedule heterogeneity for the parameters. Furthermore, it may be desirable to make the MAP prior more robust for possible unwarranted use of data from *S*_1_. To achieve this, the robust MAP prior (BVN_RMAP_) can be obtained by mixing the MAP prior (BVN_MAP_) with the WIP (BVN_WIP_) [8, 17], i.e.

$$\begin{array}{*{20}l} \text{BVN}_{\text{RMAP}} = w \, \text{BVN}_{\text{MAP}} + (1-w) \, \text{BVN}_{\text{WIP}},  \end{array} $$

where *w* is the weight which can be chosen, for example, from the range of 0.5 and 0.9. Neuenschwander et al. [8] suggested the use of *w*=0.8, and in this paper we follow their suggestion. After the robust MAP prior is derived, the BLRM is used to inform dose-escalation decisions. The R package OncoBayes2 [25] can be used to implement the BLRM and the BLRM MAP.

### The continual reassessment method (CRM)

The models described in this section follow the CRM and B-CRM described by Liu et al. [13]. For dose *d*, *p*_*d*_ is the prespecified DLT probability, also known as prior skeletons. The relationship between the prior skeletons *p*_*d*_ and DLT probabilities *π*_*d*_ are given by a power model

$$\begin{array}{*{20}l} \pi_{d} = \, p_{d}^{\exp(\alpha)} \end{array} $$

where *α* is the model parameter. For the dose-escalation decisions, the posterior mean of the *π*_*d*_ is used. The dose with posterior mean of *π*_*d*_ closest to the target probability *ϕ* is recommended for the next cohort. Once the maximum sample size is reached, the dose with posterior mean of *π*_*d*_ closest to the target probability is declared as the MTD.

Following Liu et al. [13], we use a WIP for the *α*, namely $\mathcal {N}(0, 2^{2})$. To determine the prior skeletons, we used the method developed by Lee and Cheung [26].

### The bridging CRM (B-CRM)

We now consider phase I dose-escalation trials with sequential multiple schedules. Assume that the first step of the phase I trial with schedule *S*_1_ is completed with $J_{S_{1}}$ doses, namely $\phantom {\dot {i}\!}b_{1}, b_{2}, \ldots, b_{J_{S1}}$. The first step of the trial resulted in binomial data $D_{S_{1}} = (x_{j}, m_{j})$ where *x*_*j*_ is the number of patients who experienced DLT and *m*_*j*_ is the cohort size at dose *b*_*j*_. Firstly, we can estimate the DLT probabilities using a probit model, i.e.

$$\begin{array}{*{20}l} \pi_{j}^{(P)} \equiv \, \pi^{(P)}(b_{j}) = \, \Phi(\beta_{0} + \beta_{1} \, b_{j}),  \end{array} $$

where the superscript in $\pi _{j}^{(P)}$ refers a parametric estimate; *Φ* is the cumulative distribution function of the standard normal distribution; *β*_0_ and *β*_1_ are the model parameters. Secondly, we estimate a non-parametric estimate of the DLT probabilities using isotonic regression [27]

$$\begin{array}{*{20}l} \pi_{j}^{(NP)} = \text{max}_{0 \leq u \leq j} \, \text{min}_{j \leq v \leq J_{S_{1}}} \frac{\sum_{k=u}^{v} x_{k}}{\sum_{k=u}^{v} m_{k}}.  \end{array} $$

The isotonic estimates of DLT probabilities can be obtained using the pooled-adjacent-violators algorithm [28].

In order to gain advantage of both parametric and non-parametric estimates of DLT probabilities, we use a mixture estimator of DLT probabilities:

$$\begin{array}{*{20}l} \pi_{j} = w_{j} \, \pi_{j}^{(P)} + (1-w_{j}) \, \pi_{j}^{(NP)},  \end{array} $$

where the weights *w*_*j*_ are calculated from data. The following weights are used: $w_{j} = \frac {\lambda _{j}}{\lambda _{j} + 1}$ where *λ*_*j*_ is the likelihood ratio evaluated at dose level *j* under the probit model and isotonic regression. The *λ*_*j*_ is given by

$$\begin{array}{*{20}l} \lambda_{j} = \frac{\left(\pi_{j}^{(P)} \right)^{x_{j}} \, \left(1- \pi_{j}^{(P)}\right)^{m_{j} - x_{j}}}{\left(\pi_{j}^{(NP)}\right)^{x_{j}} \, \left(1- \pi_{j}^{(NP)}\right)^{m_{j} - x_{j}}}. \end{array} $$

We estimated the DLT probabilities from the completed part of the trial involving schedule *S*_1_. The estimated DLT probabilities are used as the prior skeletons *p*_*j*_ for the analysis of next step of the phase I trial, that is involving schedule *S*_2_. Assume that there are *J* doses from schedule *S*_2_ in the next step of the trial, namely *d*_1_,*d*_2_,…,*d*_*J*_. To take into account heterogeneity in DLT probabilities between schedules, we use three sets of prior skeletons:

1. *p*_*j*_=*π*_*j*_
2. $ p_{j} = \begin {cases} & \pi _{j+1}\,\, \text {for} \,\,\, j = 1, \ldots, J - 1 \\ & \frac {\pi _{J} + 1}{2} \,\, \text {for} \,\,\, j = J \end {cases} $
3. $ p_{j} = \begin {cases} & \pi _{j-1}\,\, \text {for} \,\,\, j = 2, \ldots, J \\ & \frac {\pi _{1}}{2} \,\, \text {for} \,\,\, j = 1 \end {cases} $

The prior skeleton 1 assumes that the dose-toxicity curve obtained by the schedule *S*_1_ is same to the dose-toxicity curve of the schedule *S*_2_. Prior skeletons 2 and 3 shift the dose-toxicity curve one dose level up and one dose level down, respectively. To incorporate three different prior skeletons into the CRM model, a Bayesian model averaging approach [29] is used to estimate DLT probabilities. Then, the standard CRM is used to inform dose-escalation decisions.

For the CRM and B-CRM, the trial is terminated for safety, if the following rule is satisfied: *P*(*π*_1_>0.30)<0.90 where *π*_1_ is the DLT probability of the lowest dose. For the CRM implementation, we used the R package bcrm [30]. For the B-CRM, we use the publicly available R-code which is provided as the supplementary material of Liu et al. [13].

### TITE-PK for sequential phase I trials

TITE-PK for simultaneous investigation of multiple schedules in phase I trials were introduced in Günhan et al. [12], here we adapt it for sequential investigation of multiple schedules. The time-to-first DLT events are modeled using a time-varying (non-homogeneous) Poisson process. The hazard function is assumed to depend on an exposure measure of the drug (E(*t*)):

1 $$\begin{array}{*{20}l} h(t) = \beta \, E(t)  \end{array} $$

where *β* is the only parameter to estimate in the model.

The exposure measure is calculated using a pseudo-PK model which consists of two ordinary differential equations:

$$\begin{array}{*{20}l} &\frac{dC(t)}{dt} = - k_{e} \, C(t)\,\,\,\, \text{and} \,\,\,\, C(0) = 0  \\ &\frac{d C_{\text{eff}}(t)}{dt} = k_{\text{eff}} \, (C(t) - C_{\text{eff}}(t)) \,\,\,\, \text{and} \,\,\,\, C_{\text{eff}}(0) = 0.  \end{array} $$

where *C*(*t*) and *C*_eff_(*t*) are the concentrations of drug in the central compartment and in the so-called effect compartment, respectively. Due to non-identifiability, the volume in both compartments is set to unity by convention here. Furthermore, *k*_*e*_ is the elimination rate constant and *k*_eff_ is the PK parameter which governs the delay between the concentration in the central compartment and the concentration in the effect compartment. The parameter *k*_*e*_ is parametrized using the elimination half-life *T*_*e*_, that is $k_{e} = \frac {\text {log(2)}}{T_{e}}$. The parameters *k*_*e*_ and *k*_eff_ are assumed to be known from previous analyses, for example from another previously studied indication or pre-clinical data. In other words, the drug concentrations in the effect compartment (*C*_eff_(*t*)) is calculated by treating the PK parameters as known, following Cox et al. [31]. Thus, PK measurements are not analysed together with the toxicity data, as is done for example by Ursino et al. [32]. The model can be seen as a kinetic-pharmacodynamic model (K-PD) described in, for example, Ooi et al. [33].

TITE-PK uses an adapted EWOC criterion. For this purpose, the measure of the interest is the probability of a patient experiencing at least one DLT within the first cycle (shortly the end-of-cycle 1 DLT probability), *P*(*T*≤*t*^∗^|*C*_eff_(*t*^∗^|*d*,*f*)), where *d* and *f* refer to the dose and frequency of administration, respectively. Using basic event history analysis [34], we have the following equation

2 $$\begin{array}{*{20}l} P(T \leq t^{*}|C_{\text{eff}}(t^{*}|d,f)) = 1 - e^{-H(t^{*}|C_{\text{eff}}(t^{*}|d,f))},  \end{array} $$

which describes the relationship between the end-of-cycle 1 probabilities and the cumulative hazard function *H*(*t*). All patients without a DLT up to the end of cycle 1 will be censored at the end of cycle 1, and patients with a DLT are censored at the time of a DLT. The event indicator *δ*_*j*_ is 0 for censored events and 1 for DLT events. We can write the overall likelihood as

$$\begin{array}{*{20}l} L(T, C|\beta) = \prod_{j=1}^{J} f(T_{j}|\beta)^{\delta_{j}} \, S(C_{j}|\beta)^{(1 - \delta_{j})}  \end{array} $$

where *J* is the total number of the patients, *f*(*T*_*j*_|*β*) is the probability density function, and *S*(*C*_*j*_|*β*) is the survivor function.

Using Eq. 2, it can be shown that

3 $$\begin{array}{*{20}l} \text{cloglog}(P(T &\leq t^{*} | C_{\text{eff}}(t^{*}|d,f))) = \log(\beta) \\&+ \log(\text{AUC}_{E}(t^{*} | C_{\text{eff}}(t^{*}|d,f)))  \end{array} $$

where cloglog(*x*)=log(−log(1−*x*)) and AUC_*E*_(*t*) is the area under the curve of the exposure measure over time.

To help prior specification, *E*(*t*) is obtained by scaling *C*_eff_(*t*) using a reference schedule (reference dose *d*^∗^ and frequency *f*^∗^) at the end of the first treatment cycle (cycle 1: *t*^∗^) such that

4 $$\begin{array}{*{20}l} \text{AUC}_{E}(t^{*}| C_{\text{eff}}(t^{*}|d^{*},f^{*})) = 1.  \end{array} $$

By combining Eqs. 3 and 4, it follows that for the reference schedule cloglog(*P*(*T*≤*t*^∗^|*C*_eff_(*t*^∗^|*d*^∗^,*f*^∗^))= log(*β*). This relationship suggest to constrain *β* to be positive, which ensures that *h*(*t*)≥0, since *E*(*t*)≥0 for all *t* (see Eq. 1). The use of a reference schedules is analogous to the reference dose in the BLRM (see The Bayesian logistic regression model (BLRM) subsection). The relationship between *β* and *P*(*T*≤*t*^∗^|*C*_eff_(*t*^∗^|*d*^∗^,*f*^∗^)) helps us to specify a prior distribution for the parameter *β*. Following [8], we suggest a normal prior distribution $\mathcal {N}(\text {cloglog}(P^{*}(T \leq t^{*} | C_{\text {eff}}(t^{*}|d^{*},f^{*})), 1.25^{2}))$ for the log(*β*). Here, *P*^∗^(*T*≤*t*^∗^|*C*_eff_(*t*^∗^|*d*^∗^,*f*^∗^)) is the anticipated end-of-cycle 1 DLT probability at the reference schedule.

Similar to the BLRM, the posterior distributions of end-of-cycle 1 DLT probabilities are classified into three categories in order to inform dose-escalation decisions:

(i) *P*(*T*≤*t*^∗^|*C*_eff_(*t*^∗^|*d*,*f*))<*x* Underdosing (UD)
(ii) *x*≤*P*(*T*≤*t*^∗^|*C*_eff_(*t*^∗^|*d*,*f*))≤*y* Targeted toxicity (TT)
(iii) *P*(*T*≤*t*^∗^|*C*_eff_(*t*^∗^|*d*,*f*))>*y* Overdosing (OD)

The EWOC criterion is fulfilled, if the overdosing probability *P*(*P*(*T*≤*t*^∗^|*C*_eff_(*t*^∗^|*d*,*f*))>*y*) is smaller than the feasibility bound *a*. For the feasibility bound, we use 0.25 as in the BLRM. Analogous to the monotonicity of dose-DLT probability assumption of CRM, TITE-PK assumes the monotonicity of the exposure measure and the end-of-cycle 1 DLT probability. That is, AUC_*E*_(*t*^∗^|*C*_eff_(*t*^∗^|*d*,*f*)) is proportional to the end-of-cycle 1 DLT probabilities. Among the dose and schedule combinations which fulfill the EWOC criteria, the combination which has the lowest AUC_*E*_(*t*^∗^|*C*_eff_(*t*^∗^|*d*,*f*)) is recommended for the next cohort.

In the case of sequential investigation of multiple schedules, initially TITE-PK is used to conduct the phase I trial with *S*_1_ until the MTD is declared or trial is stopped since all doses are found to be too toxic. In this step, the frequency of administration is the same for dose-escalation decisions. Then, cohorts are recruited with Schedule *S*_2_. For dose-escalation decisions, the information from the phase I trial with *S*_1_ is treated as data together with the new information generated from the phase I trial with *S*_2_. Since TITE-PK is an exposure-response model, there is no need to re-scale the doses from different schedules to make them comparable. As opposed to BLRM MAP and B-CRM methods, data from the completed trials is treated as part of the data instead of as part of the prior distribution.

### Software implementation

We implemented TITE-PK in Stan [35] via the **rstan** R package, which employs the No-U-Turn sampler, an adaptive form of Hamiltonian Monte Carlo sampling. The No-U-Turn sampler belongs to the family of Markov chain Monte Carlo (MCMC) methods. It has been argued that the No-U-Turn sampler is more efficient and robust sampler than Gibbs sampling or Metropolis-Hastings used by WinBUGS [36] for models with complex posterior distributions [35]. For the application and simulations, four parallel chains of 1,000 MCMC iterations after warm-up of 1,000 iterations are generated. Convergence diagnostics are checked using the Gelman-Rubin statistics and traceplots in the application. There were no divergences reported for the implementation of the application. The R and Stan code to analyze the everolimus application is publicly available from Github (https://github.com/gunhanb/TITEPK_sequential). The main programming code is the Stan code from the linked folder, which conducts the Bayesian computation to calculate posterior distributions. The method can be applied by changing R-code based on the application, for example different doses or schedules, while keeping the Stan code.

### Simulation study

We compared the operating characteristics of TITE-PK and alternative methods in a simulation study. The simulation study follows the clinical scenario evaluation framework introduced by Benda et al. [37] and it is inspired by the everolimus trial. As the target probability *ϕ* for the CRM and B-CRM, we use 0.30 following Liu et al. [13]. Also, doses with DLT probabilities between 0.20 and 0.40 are considered acceptable in our simulations following Liu et al. [13]. To have a fair comparison between the methods, we use (0.20 - 0.40) to define the targeted toxicity interval of BLRM and TITE-PK, in other words *x*=0.20 and *y*=0.40 for three categories described in subsection The BLRM.

**Fig. 1:**
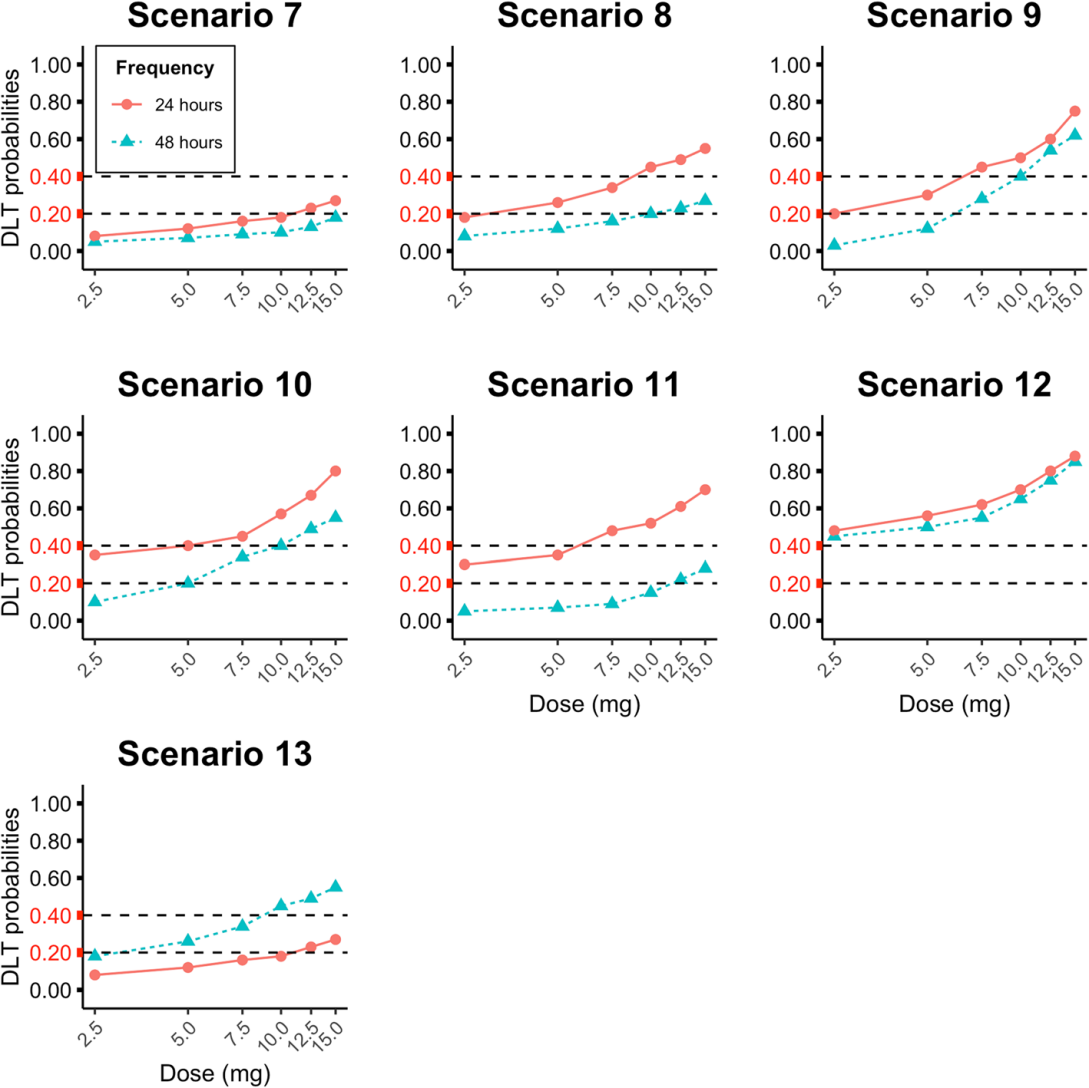
Scenarios 7-13 in the simulation study. Each scenario includes two curves of dose and DLT probabilities, which represents two schedules. Two schedules are the frequency of administration of 48 (*S*_1_) and 24 hours (*S*_2_). The horizontal dashed lines represent the boundaries of the targeted toxicity interval

Firstly, we considered scenarios only involving one schedule to compare the performance of TITE-PK to CRM and BLRM. These are Scenarios 1-6, which are listed in Table 2. Here, daily doses of 2.5, 5, 7.5, 10, 12.5, and 15 (mg) are investigated. Also, the starting dose is 2.5 mg for all methods. Secondly, we considered scenarios representing sequential phase I trials. These are Scenarios 7-13, which are listed in Table 3 and displayed in Fig. 1. Scenarios 7-13 consists of phase I trials with two steps. In the first step, doses of 2.5, 5, 7.5, 10, 12.5, 15 (mg) with the dosing frequency of 48 hours (*S*_1_) and in the second step, doses of 2.5, 5, 7.5, 10, 12.5, 15 (mg) with daily dosing (*S*_2_) are administered. There methods are assessed in Scenarios 7-13: TITE-PK, Bridging CRM (B-CRM), BLRM using MAP prior (BLRM MAP).

**Table 2:** Scenarios 1-6 in the simulation study. Doses with dose limiting toxicities in the targeted toxicity interval (0.20 - 0.40) are in boldface. Scenarios 1-6 represent phase I trials with one schedule, that is daily schedule

	Doses in mg					
Scenario	2.5	5	7.5	10	12.5	15
1	0.05	0.10	**0.20**	**0.30**	0.50	0.70
2	**0.30**	**0.40**	0.52	0.61	0.76	0.87
3	0.05	0.06	0.08	0.11	0.19	**0.34**
4	0.06	0.08	0.12	0.18	**0.40**	0.71
5	0.10	**0.22**	**0.31**	0.45	0.60	0.72
6	0.50	0.55	0.61	0.69	0.76	0.87

**Table 3:** Scenarios 7-13 in the simulation study. Daily doses with dose limiting toxicities in the targeted toxicity interval (0.20 - 0.40) are in boldface

		Doses with Schedule *S*_1_						Doses with Schedule *S*_2_					
Scenario	Schedule	2.5	5	7.5	10	12.5	15	2.5	5	7.5	10	12.5	15
7	*S*_1_	0.05	0.07	0.09	0.10	0.13	0.18						
	*S*_2_							0.08	0.12	0.16	0.18	**0.23**	**0.27**
8	*S*_1_	0.08	0.12	0.16	**0.20**	**0.23**	**0.27**						
	*S*_2_							0.18	**0.26**	**0.34**	0.45	0.49	0.55
9	*S*_1_	0.03	0.12	**0.28**	**0.40**	0.54	0.62						
	*S*_2_							**0.20**	**0.30**	0.45	0.50	0.60	0.75
10	*S*_1_	0.10	**0.20**	**0.34**	**0.40**	0.49	0.55						
	*S*_2_							**0.35**	**0.40**	0.45	0.57	0.67	0.80
11	*S*_1_	0.05	0.07	0.09	0.15	**0.22**	**0.28**						
	*S*_2_							**0.30**	**0.35**	0.48	0.52	0.61	0.70
12	*S*_1_	0.45	0.50	0.55	0.65	0.75	0.85						
	*S*_2_							0.48	0.56	0.62	0.70	0.80	0.88
13	*S*_1_	0.18	**0.26**	**0.34**	0.45	0.49	0.55						
	*S*_2_							0.08	0.12	0.16	0.18	**0.23**	**0.27**

The DLT probabilities of the doses in the simulations are determined to reflect clinically relevant settings. Scenario 1 corresponds to a phase I trial which does not include any dose with DLT probability in the overdosing interval. However, all doses are in the overdosing interval in Scenario 6. In Scenarios 2-5, different dose levels are chosen as the doses within the targeted toxicity interval (0.20 - 0.40) (see Table 2). In Scenarios 2 and 5, lower dose levels are within the targeted toxicity interval. In Scenarios 3 and 4, higher dose levels are within the targeted toxicity interval. In Scenario 7, there is no dose from Schedules 1 and 2 in the overdosing interval, whereas all doses from Schedules 1 and 2 are in the overdosing interval in Scenario 12. In Scenarios 8-10, lower dose levels of Schedule 2 are within the targeted toxicity interval (Table 3). In Scenarios 7 and 13, higher dose levels of Schedule 2 are within the targeted toxicity interval. Scenario 11 is a scenario in which the discrepancy of dose-toxicity curve between the schedules is higher than other scenarios (see Fig. 1). Scenario 13 is Scenario 8 with DLT probabilities for Schedules 1 and 2 switched. Hence, the monotonicity assumption of the exposure and DLT probabilities is violated in Scenario 13. In other words, for the same dose, toxicity is higher with the lower frequent administration.

For TITE-PK, we need to determine PK parameters. By mimicking the everolimus trial, PK parameters are chosen as follows. The elimination rate constant is taken as $k_{e} = \frac {\text {log}(2)}{30}$ (1/h). For *k*_eff_, an estimate is derived using the cycle length and the absorption rate. Specifically, a log-normal distribution is constructed by matching the inverse of cycle length 1/504 (1/h) and the absorption rate 2.5 (1/h) as the 0.025 and 0.975 quantiles, respectively. This gives a log-normal distribution with mean parameter 0.37, hence we assume that log(*k*_eff_) = 0.37.

Prior skeletons and distributions are constructed so that prior DLT probabilities from different methods are similar. For TITE-PK model, reference dose and reference dosing frequency are determined using 7.5 mg (*d*^∗^=7.5 mg) and 24 hours (*f*^∗^=1/24 1/h). A normal weakly informative prior (WIP) is chosen such that log(*β*) ∼$\mathcal {N}(\text {cloglog}(P(T \leq t^{*}) = 0.30), 1.25^{2})$. This implies that prior median of DLT probability at the reference dose and frequency is 0.30. For BLRM MAP, we choose a WIP assuming median DLT probability of 0.30 at dose 7.5 mg. More specifically, we choose a bivariate normal distribution (log(*α*_1_), log(*α*_2_))∼BVN(**m**,*Σ*) with means *m*_1_=logit(0.30) and *m*_2_=0, standard deviations *σ*_1_=2 and *σ*_2_=1, and correlation *ρ*=0. For the CRM, the prior skeleton is calculated using the method of Lee and Cheung [26] assuming an indifference interval of 0.10, which produces (0.02, 0.12, 0.30, 0.50, 0.68, 0.80). A normal prior with mean 0 and standard deviation 2 is used as the prior for the power parameter *α* in the CRM and B-CRM ($\alpha \sim \mathcal {N}(0, 2^{2})$), as suggested by Liu et al. [13].

The following simulation settings and decision rules are used for TITE-PK, BLRM and BLRM MAP. The maximum number of patients per trial was set to 60. If all doses are in the overdosing interval based on the EWOC criterion, the trial is stopped without selecting any dose as the MTD. Otherwise, the trial continues until the recommendation of the MTD. The recommended MTD must meet the following conditions:

(i) At least 6 patients have been treated at the MTD.
(ii) A minimum of 21 patients have already been treated in the trial.

For the CRM and B-CRM, the trial is terminated for safety, if the following rule is satisfied: *P*(*π*_1_>0.30)<0.90 where *π*_1_ is the DLT probability of the lowest dose. The sample size of 21 patients is used unless the trial is stopped due to the safety. For all methods in the simulations, cohort sizes of 3 are used and data for 1,000 trials were generated per scenario.

## Results

### Simulation results

The simulation results for Scenarios 1-6 (see Table 2) are summarized in Table 4. We calculated six different metrics to evaluate the performance of different methods. Scenarios 1-6 represent phase I trials with one schedule investigated. In Scenario 1, TITE-PK slightly outperforms other methods in terms of recommending the MTD in the targeted toxicity interval. The corresponding percentages are 78% for TITE-PK, 75% for BLRM and 73% for CRM. Also, BLRM yields slightly lower percentage for the MTD selection in the overdosing interval compared to TITE-PK and CRM. BLRM selects the MTD in the overdosing interval in 6% of the time, while TITE-PK and CRM do this in 11% and 9% of the time, respectively. In Scenario 2, CRM yields higher percentage for the MTD selection in the targeted toxicity interval compared to the TITE-PK and BLRM. CRM recommends the MTD in the targeted toxicity interval in 61% of the time, while TITE-PK and BLRM do this in 52% and 49% of the time, respectively. Three methods perform similarly in terms of recommending the MTD in the overdosing interval. In scenario 3, TITE-PK results in the best performance in terms of the MTD selection in the targeted toxicity interval. TITE-PK recommends the MTD in the targeted toxicity interval 75% of the time, while BLRM and CRM do this in 64% and 24% of the time, respectively.

**Table 4:** Simulation results for TITE-PK, CRM, and BLRM in Scenarios 1-6

	Scenario					
	1	2	3	4	5	6
Probability of selecting MTD in the targeted toxicity interval						
TITE-PK	0.78	0.52	0.75	0.36	0.71	n/a
CRM	0.73	0.61	0.24	0.22	0.79	n/a
BLRM	0.75	0.49	0.64	0.14	0.78	n/a
Probability of selecting MTD in the overdosing interval						
TITE-PK	0.11	0.03	n/a	0.06	0.17	0.11
CRM	0.09	0.04	n/a	0.04	0.10	0.14
BLRM	0.06	0.02	n/a	0.04	0.10	0.07
Probability of selecting no combination as MTD						
TITE-PK	0.01	0.42	0.00	0.01	0.04	0.87
CRM	0.01	0.36	0.01	0.01	0.03	0.86
BLRM	0.01	0.48	0.01	0.01	0.04	0.92
Mean number of patients enrolled						
TITE-PK	24.7	15.4	23.3	27.0	22.8	8.1
CRM	20.9	15.7	20.9	20.8	20.5	8.9
BLRM	23.6	14.9	24.2	24.8	21.9	7.3
Proportion of patients enrolled in the overdosing interval						
TITE-PK	0.28	0.15	n/a	0.13	0.27	1.00
CRM	0.05	0.05	n/a	0.01	0.06	1.00
BLRM	0.10	0.08	n/a	0.11	0.11	1.00
Proportion of DLT observed						
TITE-PK	0.28	0.38	0.21	0.25	0.30	0.52
CRM	0.18	0.33	0.11	0.15	0.22	0.51
BLRM	0.21	0.35	0.15	0.20	0.24	0.50

In scenario 4, all methods perform poorly in terms of selecting the MTD in the targeted toxicity, while TITE-PK results in the best performance. TITE-PK yields 36% percentage for the MTD selection in the targeted toxicity interval, while CRM and BLRM yields 22% and 14%, respectively. In scenario 5, CRM (79%) and BLRM (78%) produces slightly higher percentages than TITE-PK (71%) in terms of the selecting MTD in the targeted toxicity interval. In scenario 6, all doses are in the overdosing interval. BLRM (92%) stops the trial with slightly higher percentages compared to CRM (86%) and TITE-PK (87%).

In Scenarios 1, 3, 4 and 5, TITE-PK and BLRM enrolls slightly higher number of patients and results in slightly higher proportions of DLT observed in comparison to CRM. Overall, none of the methods shows superior performance in terms of the investigated metrics. The results depend on the scenarios. Similar results from the comparison of BLRM and CRM was also obtained by the simulation studies in Neuenschwander et al. [4].

We continue with Scenarios 7-13 in which sequential phase I trials are investigated. The simulation results under Scenarios 7-13 (see Table 3) are summarized in Table 5. In Scenario 7, BLRM MAP produces the best performance in terms of the MTD selection in the targeted toxicity interval, while TITE-PK is the second. The corresponding percentages are 95%, 90%, and 83% for BLRM MAP, TITE-PK, and B-CRM respectively. In Scenarios 8-11, TITE-PK demonstrates superior performance in terms of selecting the MTD in the targeted toxicity interval. TITE-PK selects the MTD in the targeted toxicity interval in 14%, 17%, 16%, and 10% more simulated trials in comparison to the second best performed method in Scenarios 8-11, respectively. In Scenarios 8 and 9, TITE-PK produces lower percentages in terms of the MTD selection in the overdosing interval, selecting MTD in 16% and 3% less simulated trials compared to BLRM MAP. In Scenario 11, CRM (28%) displays superior performance in terms of the MTD selection in the overdosing interval in comparison to other methods. In Scenario 12, TITE-PK and BLRM MAP displays better performance than B-CRM by stopping the trial in 98% and 97% of the time, while requiring less patients than other methods. The monotonicity assumption of the exposure and DLT probabilities is violated in Scenario 13. In Scenario 13, B-CRM outperforms other methods by selecting MTD in the targeted toxicity interval in 22% more trials compared to the BLRM MAP. TITE-PK (17%) displays the worst performance in terms of the MTD selection in the targeted toxicity interval.

**Table 5:** Simulation results for TITE-PK, B-CRM, and BLRM-MAP in Scenarios 7-13

	Scenario						
	7	8	9	10	11	12	13
Probability of selecting MTD in the targeted toxicity interval							
TITE-PK	0.90	0.70	0.94	0.84	0.62	n/a	0.17
B-CRM	0.83	0.50	0.64	0.60	0.52	n/a	0.77
BLRM MAP	0.95	0.56	0.77	0.68	0.46	n/a	0.55
Probability of selecting MTD in the overdosing interval							
TITE-PK	n/a	0.22	0.05	0.02	0.37	0.02	n/a
B-CRM	n/a	0.38	0.08	0.00	0.28	0.25	n/a
BLRM MAP	n/a	0.40	0.21	0.10	0.41	0.03	n/a
Probability of selecting no combination as MTD							
TITE-PK	0.00	0.02	0.01	0.14	0.00	0.98	0.15
B-CRM	0.00	0.02	0.02	0.28	0.20	0.75	0.00
BLRM MAP	0.00	0.02	0.02	0.22	0.12	0.97	0.01
Mean number of patients enrolled							
TITE-PK	21.7	21.7	21.4	19.4	21.8	3.7	19.7
B-CRM	21.0	21.0	21.0	18.0	19.0	9.0	21.1
BLRM MAP	21.5	23.6	21.6	20.0	22.8	4.8	23.4
Proportion of patients enrolled in the overdosing interval							
TITE-PK	n/a	0.39	0.17	0.12	0.61	1.00	n/a
B-CRM	n/a	0.46	0.15	0.06	0.72	1.00	n/a
BLRM MAP	n/a	0.59	0.40	0.26	0.70	1.00	n/a
Mean number of DLT observed							
TITE-PK	5.3	8.2	6.2	7.5	10.2	1.8	2.4
B-CRM	4.5	7.0	7.3	7.5	8.5	4.0	3.0
BLRM MAP	5.7	9.7	7.7	8.2	11.1	2.4	3.9

The number of people required in a trial is very crucial measure to assess the performance of a method. Table 5 displays the mean number of people required in a trial for the investigated methods and the lower is more desirable. In Scenarios 7-13 except 12, TITE-PK and B-CRM enrolls similar number of patients, whereas BLRM MAP requires slightly higher number of patients. In Scenario 12, TITE-PK and BLRM MAP requires lower number of patients in comparison to B-CRM. In Scenarios 7-13 except 12, in terms of the proportion of DLT observed, all methods perform similarly. In Scenarios 7-12, TITE-PK displays the best or the second best performance in terms of the MTD selection in the targeted toxicity and overdosing intervals. However, TITE-PK clearly shows poor performance in Scenario 13, which is expected, as the monotonicity assumption between exposure and DLT probability is violated.

### Revisiting the everolimus trial

Returning to the data set described before, consider the everolimus trial shown in Table 1. Firstly, we analyse the data only from the daily schedule using the BLRM, the CRM, and the TITE-PK. Secondly, we analyse it as if the trial is conducted sequentially, specifically *S*_1_ is weekly schedule and *S*_2_ is daily schedule using BLRM MAP, B-CRM, and TITE-PK. The reference schedule is determined using dosing amount of 5 mg (*d*^∗^=5 mg) and dosing frequency of 24 hours (*f*^∗^=1/24 1/h). For TITE-PK, PK parameters are chosen such that *T*_*e*_=30 (hours) and log(*k*_eff_)=0.37 as explained in the simulation study.

**Fig. 2:**
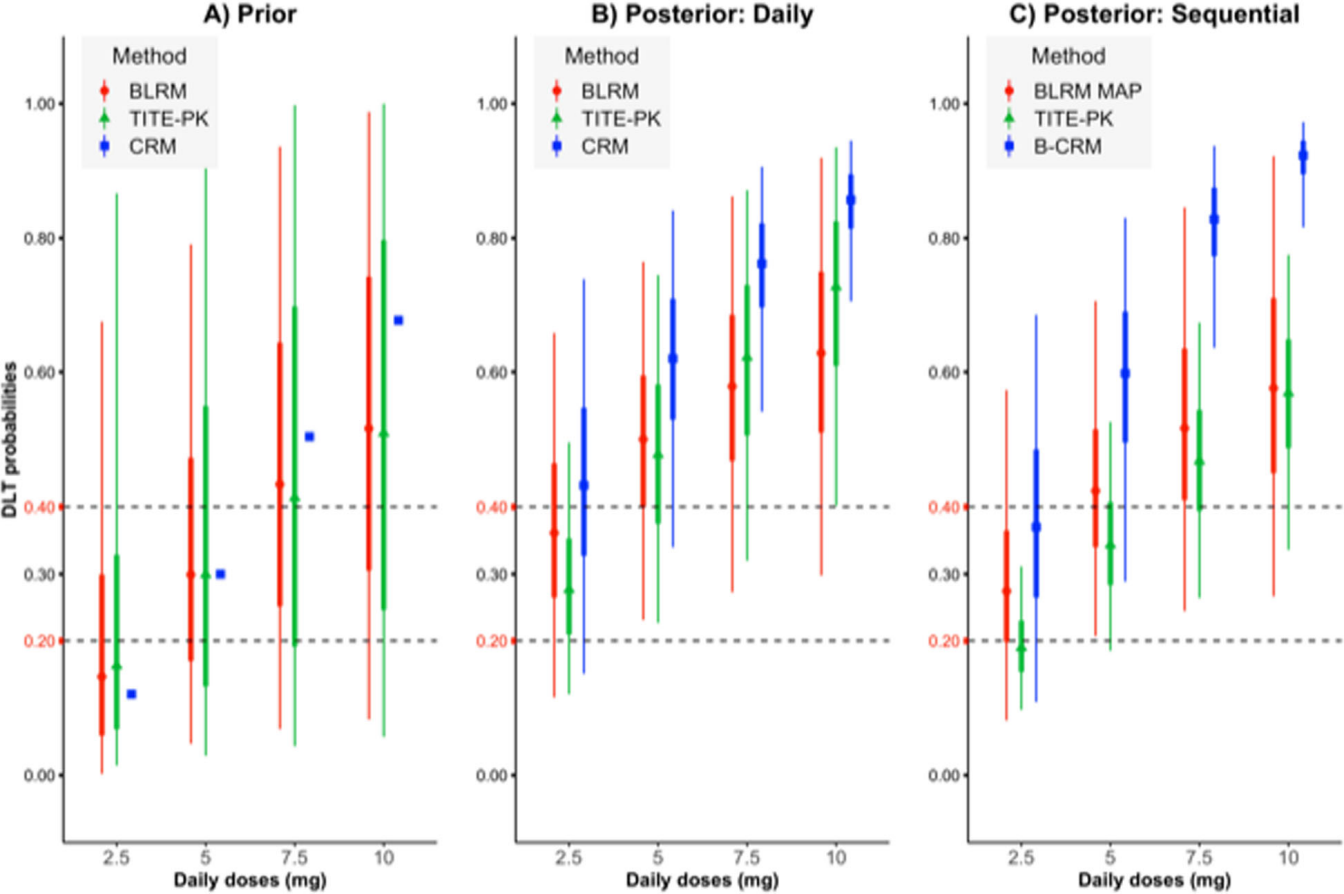
Everolimus trial Prior medians **A**, posterior medians daily **B**, and sequential **C**, 50% equi-tailed credible intervals (thick lines), and 95% equi-tailed credible intervals (thin lines) of daily doses for DLT probabilities obtained by BLRM (BLRM-MAP for Sequential), CRM (B-CRM for Sequential), and for end-of-cycle 1 DLT probabilities obtained by TITE-PK. Prior skeletons are shown for CRM in the plot A. “Sequential” refers that analysis is done by assuming the trial is conducted sequentially, namely firstly weekly schedule, secondly daily schedule. Also, “Daily” means data only from daily schedule is considered. Vertical dashed lines (0.20-0.40) are the boundaries of the targeted toxicity interval

To compare BLRM, CRM and TITE-PK models, priors are constructed so that prior DLT probabilities are similar. To define a WIP for BLRM, we choose a bivariate normal prior with following parameters $\phantom {\dot {i}\!}(m_{1} = \text {logit}(\pi _{d^{\ast }} = 0.30), m_{2} = 0, \sigma _{1} = 1.25, \sigma _{2} = 1, \rho = 0)$. For the CRM, we use the target probability of 0.30. The prior skeleton is, then, calculated assuming an indifference interval of 0.10, which produces (0.12, 0.30, 0.50, 0.68). For TITE-PK, a normal WIP is chosen such that $\log (\beta) \sim \mathcal {N}(\text {cloglog}(P(T \leq t^{*}) = 0.30), 1.25^{2})$ at the reference dose and schedule. The summaries of prior DLT probabilities of BLRM and TITE-PK, and prior skeletons of CRM are shown in Fig. 2A. Points, thick lines and thin lines correspond to median estimates, the 50% and the 95% equi-tailed credible intervals, respectively. Vertical dashed lines (0.20-0.40) are the boundaries of the targeted toxicity interval. Recall that, in TITE-PK and BLRM, eligible doses are determined based on the EWOC criterion, whereas CRM selects the dose closest to the target probability.

Figure 2B displays the posterior estimates of DLT probabilities, when we only consider daily schedule data. BLRM suggests that all doses are in the overdosing interval, meaning that the trial should be stopped without any dose declared as the MTD. The estimated overdosing probability of 2.5 mg is 0.40, which is higher than 0.25. For TITE-PK, only 2.5 mg is not in the overdosing interval. The overdosing probability of 2.5 mg is 0.14, *P*(*P*(*T*≤*t*^∗^|*d*=2.5,*f*=24)>0.40)=0.14, which is smaller than 0.25. Although median DLT probability estimate of CRM is higher than the median DLT probability estimate of BLRM, CRM does not conclude that the trial should be stopped. This is because, *P*(*π*_1_>0.30)=0.80, which is smaller than 0.90. Furthermore, credible intervals obtained by the CRM is getting shorter with the increasing dose, which was also observed by Neuenschwander et al. [4]. Overall, high overdosing probabilities for all doses seem reasonable, since 2 DLT were observed in the 4 patients with 2.5 mg, and 3 DLT were in the 6 patients with 5 mg dose.

We continue by treating the data from the weekly schedule as the completed trial in a sequential phase I trial. We estimate the DLT probabilities of daily doses, but also taking into consideration the data coming from the weekly data. To implement BLRM-MAP, the MAP prior is calculated based on the weekly data. Later, the BLRM is fitted and posterior estimates of DLT probabilities are obtained. In the B-CRM, prior skeletons are calculated using the weekly data. Then, CRM via a Bayesian model averaging method is used to estimate DLT probabilities. TITE-PK, naturally, combines information from different schedules. Figure 2C displays the estimated posterior summaries of DLT probabilities of daily doses obtained by TITE-PK, BLRM-MAP and B-CRM approaches. For both TITE-PK and BLRM-MAP, the overdosing probability of dose 2.5 mg is decreased substantially, namely from 0.40 to 0.18 for BLRM-MAP, and from 0.14 to 0.00 for TITE-PK. For CRM, the probability *P*(*π*_1_>0.30) is also decreased from 0.80 to 0.67. The reduction of the overdosing probabilities of 2.5 mg seems reasonable, since in the weekly schedule data, no DLT were observed in the 5 patients with 20 mg and 4 DLT were in the 13 patients with 30 mg. The interval estimates of 2.5 mg and 5 mg obtained by TITE-PK are shorter, hence more precise estimates compared to BLRM-MAP and B-CRM. All three methods suggest that daily 2.5 mg is sufficiently safe, hence it can be declared as the MTD which was the conclusion of the original phase I trial.

**Fig. 3:**
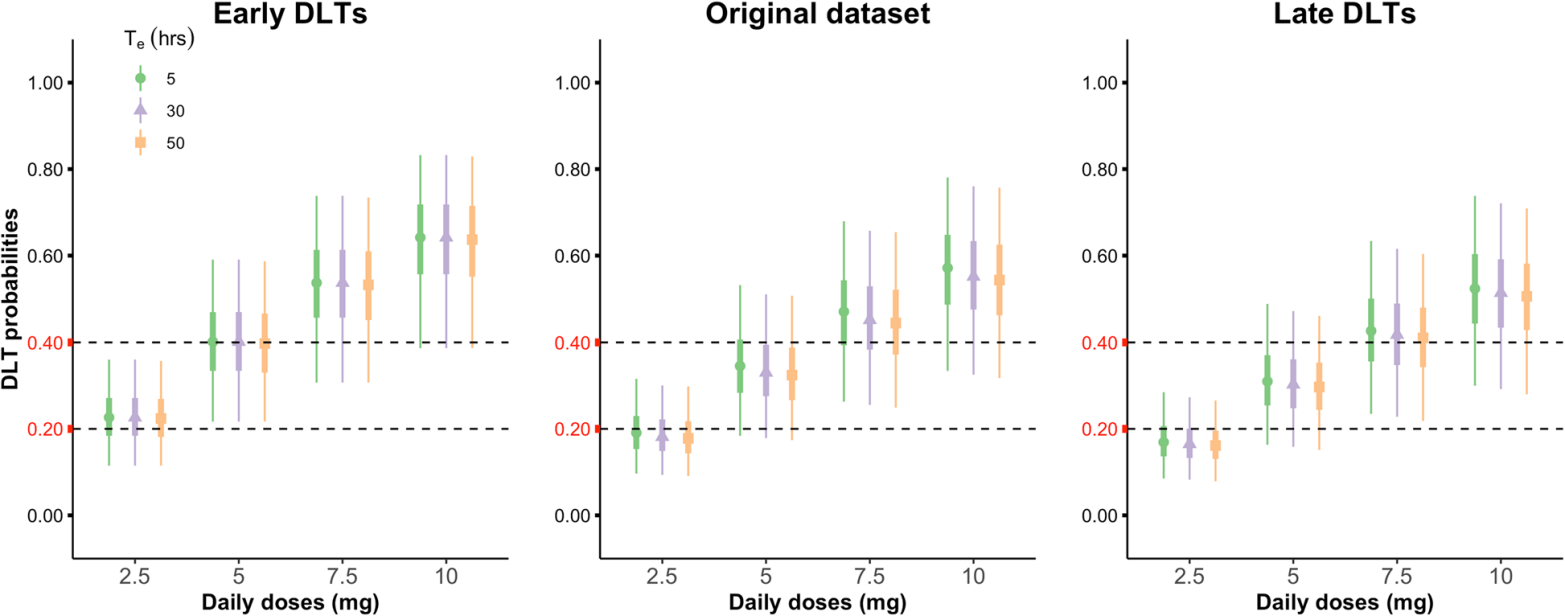
Misspecification of elimination half-life *T*_*e*_ and different timing of DLT. Using different values of *T*_*e*_, posterior median, 50% and 95% equi-tailed credible intervals for end-of-cycle 1 DLT probabilities obtained by TITE-PK for two hypothetical datasets (early DLT and late DLT) and the original everolimus trial dataset are shown. Early DLT dataset and late DLT dataset are created by changing timing of DLT from day 15 to day 1.5 and to day 20.5, respectively. Data from both weekly and daily schedules are included in the analysis

As pointed out in Methods section, by construction of TITE-PK, the elimination half-life *T*_*e*_ is treated as known. To investigate the influence of misspecification of the *T*_*e*_ parameter, we fit TITE-PK using *T*_*e*_ ranging from 5 to 50 hours. The timing of all DLT (in total 9 DLT) were reported at day 15. To examine what would be the influence of the timing of DLT, we also fit TITE-PK to two hypothetical datasets. Early DLT dataset and late DLT dataset are created by changing timing of DLT from day 15 to day 1.5 and to day 20.5, respectively. Posterior estimates of DLT probabilities for different *T*_*e*_ values and for different timing of DLT are shown in Fig. 3. The middle plot corresponds to the original everolimus trial data. Firstly, the posterior medians and credible intervals obtained by different *T*_*e*_ values look very similar. In practice, a reliable estimate of elimination half-life is often not available. Hence, these results are reassuring for the practicality of TITE-PK. Secondly, timing of DLT has a crucial affect on the posterior estimates, and hence the overdosing probabilities. Having the same number of DLT, the earlier the DLT happened, the higher the overdosing probability of the corresponding dose estimated. This makes sense, since one would expect the drug to be more toxic if DLT happened earlier than later.

## Discussion

In this manuscript, we considered a sequential trial in which trial with schedule *S*_1_ is already completed. Another type of a sequential trial can be designed to use the so-called concurrent co-data [14]. That is, the trial with Schedule *S*_1_ is still ongoing, and we would like to utilize the information from the Schedule *S*_1_ to inform dose-escalation decisions with Schedule *S*_2_ (and vice versa). TITE-PK can be used for such designs as well. We did not investigate these situations, since these are beyond the scope of the paper.

In a sequential phase I trial, strata sometimes refer to other than schedules, e.g. patient populations. In such situations, the integration of different strata can be achieved using a MAP approach. Since TITE-PK is parametrized by mimicking the interpretable parameters of the BLRM, it can be extended to use a MAP approach like the BLRM. A key strength of the TITE-PK approach is its ability to integrate the data from different treatment schedules in a model based approach. This makes ad-hoc approaches like dose re-scaling obsolete which reduces the need for strong discounting of historical data from different schedules. However, discounting may still be needed to account for other sources like different patient populations. Recently, Li and Yuan [16] introduced a method to find the MTD for paediatric dose-escalation trial by incorporating information from the concurrent adult data. Their method is based on the CRM and uses Bayesian model averaging to control discounting from the adult data. The BLRM MAP approach makes the assumption of the exchangeability between different schedules. Instead of using a MAP prior, one can use exchangeability/non-exchangeability (EX-NEX) [25, 38] approach for phase I trials with multiple schedules, which relaxes the exchangeability assumption.

The monotonicity assumption of the exposure and DLT probabilities is often very reasonable but could be considered a limitation of TITE-PK. Similarly, the BLRM and the CRM assumes the monotonicity of the doses and DLT probabilities. Since, we have used a linear PK model within TITE-PK, the monotonicity of the exposure and DLT probabilities implies the monotonicity of the dose and DLT probabilities.

The main purpose of the pseudo-PK model is to account for the dose and schedule (frequency of administration). This is done in a relatively approximate way and these parts of the model can be improved in a future work. We see a number of challenges about expanding the model to also include real PK data and a realistic PK model. An important challenge is operational, that is PK data is commonly only available with some delay as compared to DLT data. Furthermore, coupling the PK with the PD model leads to challenges implied by joint models which need to be addressed (like consequences of model misspecification in either model). However, one can consider alternative PK models, for instance a first-order absorption linear one compartment model instead of the described pseudo-PK model.

In the simulations where we investigated phase I trials with one schedules (Scenarios 1-6), we assumed the monotonicity of dose and DLT probabilities. When there is a heavy violation of the assumption of the monotonicity (as in Scenarios 13), the operating characteristics are expected to be weaker compared to bridging CRM or BLRM MAP. The violation of the assumptions occurred, since there is a clear conflict in exposure and DLT profiles between different schedules. Such violations can be informed using the external PK data from the ongoing trial. An extension combining TITE-PK with MAP could be more useful for such situations.

## Conclusions

We have adapted TITE-PK for efficiently estimating the maximum tolerable dose in sequential phase I trials involving multiple schedules. To integrate data from different schedules, TITE-PK makes use of exposure-response modelling considering kinetic drug properties. Moreover, we have demonstrated that TITE-PK can be used as an alternative to the standard methods like the BLRM or CRM to conduct phase I trials with only one schedule. In these trials, we have demonstrated that TITE-PK displays similar performance compared to CRM and BLRM. In scenarios with sequential phase I trials, TITE-PK mostly displays superior performance in terms of acceptable dose recommendations in comparison to the bridging CRM and BLRM using MAP approach. An application involving weekly and daily schedules is used to illustrate TITE-PK. Also, using the application, we have shown that TITE-PK is robust against the misspecification of the PK parameter elimination half-life.

## Data Availability

The everolimus dataset used in the paper is available from Besse et al. [21].

## Abbreviations

CRM: Continual Reassessment Method
BLRM: Bayesian Logistic Regression Model
PK: Pharmacokinetics
TITE-PK: Time-to-event Pharmacokinetic Model
EWOC: Escalation with Overdose Control
MTD: Maximum Tolerated Dose
DLT: Dose-Limiting-Toxicity
MAP: Meta-Analytic-Predictive
B-CRM: Bridging Continual Reassessment Method
EX-NEX: Exchangeability/Non-Exchangeability
